# IKZF1基因改变在急性B淋巴细胞白血病中的预后意义及移植策略选择

**DOI:** 10.3760/cma.j.cn121090-20250826-00400

**Published:** 2026-05

**Authors:** 潇潇 马, 尔烈 姜

**Affiliations:** 1 中国医学科学院血液病医院（中国医学科学院血液学研究所），血液与健康全国重点实验室，国家血液系统疾病临床医学研究中心，细胞生态海河实验室，天津 300020 State Key Laboratory of Experimental Hematology, National Clinical Research Center for Blood Diseases, Institute of Hematology & Blood Diseases Hospital, Chinese Academy of Medical Sciences & Peking Union Medical College, Haihe Laboratory of Cell Ecosystem, Tianjin 300020, China; 2 天津医科大学，天津 300070 Tianjin Medical University, Tianjin 300070, China

## Abstract

Ikaros家族锌指蛋白1（IKZF1）基因改变是急性B淋巴细胞白血病（B-ALL）中常见的遗传学异常，与患者复发风险增加及不良预后密切相关。本文系统阐述了IKZF1基因的生物学功能及其不同突变类型的临床意义，并以此为基础，构建了一个从诊断到移植后管理的全程临床决策框架。文章剖析了免疫治疗作为移植“桥梁”的核心价值，提出了以“微小残留病（MRD）清除动力学”为指导的移植方案抉择模型（异基因对自体），并明确了移植后基于MRD的主动干预策略。

## Abstract (en)

Gene alterations in Ikaros family zinc finger protein 1（IKZF1）are common genetic abnormalities in B-cell acute lymphoblastic leukemia（B-ALL）and are closely associated with an increased risk of relapse and poor prognosis. In this study, the biological functions of the IKZF1 gene and the clinical significance of its various mutation types were systematically elaborated. Accordingly, a comprehensive clinical decision-making framework spanning from diagnosis to post-transplantation management was constructed. This framework provides an in-depth analysis of the pivotal role of immunotherapy as a “bridge” to transplantation, innovatively proposes an “MRD clearance kinetics”-guided model for deciding on transplantation modalities（allogeneic versus autologous）, and defines proactive post-transplantation intervention strategies based on MRD monitoring. Furthermore, this review aims to provide a clear and actionable blueprint for individualized therapy for B-ALL patients with IKZF1 alterations.

急性淋巴细胞白血病（ALL）是一种起源于淋巴祖细胞的恶性克隆性疾病，以骨髓中未成熟淋巴细胞大量增殖和累积为主要特征[1]。根据免疫表型特征，ALL可分为B细胞系（B-ALL）和T细胞系（T-ALL），其中B-ALL约占成人ALL的75％[2]。尽管儿童ALL的治愈率已超过90％，成人B-ALL的预后仍较差，5年总生存率30％～40％[3]。这种差异与成人患者遗传学异常更为复杂、治疗抵抗性更强密切相关。其中，IKZF1基因的改变作为最常见的遗传学异常之一，不仅是独立的预后不良因素，更在疾病复发、治疗抵抗机制中扮演核心角色，直接影响治疗策略的制定，尤其是移植方案的选择[4]–[5]。本文旨在通过整合IKZF1的生物学特性、可检测残留病（MRD）动态监测及新型治疗进展，构建以临床决策为核心的论述体系，并重点探讨免疫治疗如何为高危患者创造移植机会，治疗反应（特别是MRD动态）如何指导移植策略的动态调整，移植后应如何实施主动管理以克服高复发倾向，以期为伴IKZF1改变的B-ALL患者的个体化精准治疗提供理论依据与实践指导。

一、Ikaros蛋白的生物学功能及IKZF1基因突变形式

1. IKZF1基因的生物学特性：IKZF1基因位于染色体7p12.2区域，全长约90 kb，包含8个外显子，编码由519个氨基酸组成的Ikaros蛋白[6]。该蛋白属于锌指转录因子家族，其N末端的DNA结合域由4个锌指结构组成，C末端的蛋白质互作域含2个锌指结构[7]。借助锌指结构，Ikaros可特异性结合DNA序列，调控下游靶基因的转录，参与B细胞受体信号传导、细胞周期及凋亡等关键生物学过程[8]。

在淋巴细胞正常发育中，Ikaros通过调控PU.1、IRF4、BCL6等关键转录因子表达，精确引导淋巴祖细胞向B细胞分化[9]。此外，Ikaros还可通过组蛋白修饰与染色质重塑等表观遗传机制，影响基因可及性与表达水平，进而调控淋巴细胞发育进程[10]。

2. IKZF1改变的分子机制：ALL中IKZF1改变主要包括基因缺失、点突变及异常剪接等形式[11]。缺失是最常见类型，可分为全基因缺失、部分外显子缺失或亚显微缺失等；点突变多集中于锌指区，损害DNA结合能力[12]；异常剪接则导致功能域缺失的异构体，多具显性负效应[13]。上述变异导致Ikaros功能丧失或显性负效应，破坏其正常转录调控功能。功能丧失型突变使IKZF1的DNA结合能力下降，而显性负效应突变体可通过与野生型蛋白竞争性结合DNA或形成无功能二聚体，干扰正常功能[14]，最终引发分化阻滞、增殖失控与凋亡抵抗。

3. IKZF1改变与其他遗传学异常的协同作用：IKZF1改变常与CDKN2A/B缺失、PAX5变异、JAK-STAT通路突变等共存，形成特定分子亚型[15]。这些异常的协同效应加剧疾病恶性进展与治疗抵抗。研究显示，IKZF1异常与JAK-STAT信号通路激活存在功能关联：异常Ikaros蛋白无法有效抑制JAK-STAT通路，导致持续激活，促进白血病细胞增殖[16]。同时，IKZF1改变常与CRLF2重排共存，共同驱动Ph样B-ALL发生[17]。此类多基因协同模式为理解B-ALL分子机制提供了新视角，也为联合靶向治疗策略开发奠定基础。

二、IKZF1改变的临床意义及预后价值

1. IKZF1改变的流行病学特征：IKZF1改变发生率因年龄与遗传背景而异，儿童B-ALL中为15％～20％，成人中达30％～40％[18]，反映成人患者遗传学复杂性更高。该改变发生率也与特定遗传亚型紧密相关：Ph阳性B-ALL中可达70％～80％[19]；Ph样B-ALL中为50％～60％[20]；其他亚型中通常低于20％。提示IKZF1改变在特定亚型发病机制中可能起关键作用。

2. IKZF1改变的预后价值：多项研究证实IKZF1改变是B-ALL预后不良的独立危险因素。Mullighan等[21]通过全基因组分析首次报道IKZF1缺失在B-ALL中发生率高达28.6％，且与复发风险增加和生存率下降显著相关，后续研究在不同年龄组和遗传亚型中验证了这一结论。儿童B-ALL中，IKZF1改变与较高复发风险和较低生存率相关。一项针对儿童Ph^+^ B-ALL的研究显示，IKZF1缺失发生率高达66％，且无论是否使用酪氨酸激酶抑制剂，IKZF1缺失患者的无病生存率均显著低于野生型患者[22]。Tang等[23]针对164例成人B-ALL患者的研究显示，IKZF1突变是独立预后不良因素，且BCR::ABL阳性亚组预后更差；allo-HSCT可显著改善IKZF1突变患者的生存率。

3. IKZF1改变类型的预后差异：近年研究发现，不同IKZF1改变类别可能具有不同预后意义。例如，显性负效应突变较单倍体不足突变可能导致更差的临床结局[24]；全长缺失较部分外显子缺失具更强预后预测价值[25]。提示未来需对IKZF1改变进行精细分类，以更准确评估预后并指导治疗。同时，IKZF1改变与其他遗传异常的组合模式也可能影响预后，需大样本研究进一步验证。

三、MRD在伴IKZF1基因改变B-ALL预后评估中的价值

1. MRD的检测方法与技术进展：MRD指治疗后体内残留的少量白血病细胞，是B-ALL最重要的预后因素之一[26]。目前国际常用MRD检测方法主要包括多参数流式细胞术（MFC）和聚合酶链反应（PCR）技术。需要明确的是，IKZF1缺失作为一种基因变异，其本身需要通过荧光原位杂交（FISH）、基因芯片或二代测序（NGS）等分子遗传学方法进行诊断。而“IKZF1 plus”是一个高危分子亚型的概念，特指IKZF1缺失伴其他高危异常（如CDKN2A/B、PAX5缺失）的群体。临床常规的MRD监测（MFC、靶向PCR）旨在评估疾病负荷，并不直接检测IKZF1状态。因此，实践中应是先通过遗传学方法诊断“IKZF1 plus”高危亚型，再利用MRD技术对其进行精细的疗效监控。因此，IKZF1定义了“谁”是最高危的患者，而MRD则告诉我们这位高危患者“当前”的治疗反应如何，二者从不同维度共同构成了风险适应的个体化治疗基石。

MFC通过检测白血病细胞特异免疫表型，灵敏度可达10^−4^～10^−5^［27]，优势在于快速、应用范围广，但需专业分析经验与标准化流程。PCR技术包括基于抗原受体基因重排的PCR与实时定量PCR（qPCR），灵敏度达10^−4^～10^−6^［28]。近年来，数字PCR（dPCR）与NGS技术进一步提升了MRD检测灵敏度与特异性，尤其NGS可同时追踪多个克隆，为MRD监测提供了新工具[29]。

2. MRD的临床意义：多项研究证实诱导治疗结束后MRD水平与复发风险显著相关。Bassan等[30]的Meta分析显示，MRD阳性是ALL患者无事件生存和总生存的强有力独立预后因素。

MRD动态监测对指导B-ALL治疗决策至关重要。美国国家综合癌症网络（NCCN）指南与欧洲白血病网络（ELN）指南均推荐将MRD（MFC或qPCR，灵敏度10^−4^～10^−5^）状态作为B-ALL风险分层与治疗选择的重要依据[31]–[32]。早期MRD清除（如诱导治疗结束后）提示良好预后，而持续MRD阳性或MRD转阳则需强化治疗或考虑移植[33]。

3. IKZF1改变与MRD的关联：值得注意的是，IKZF1改变与MRD状态密切相关。研究显示伴IKZF1改变的B-ALL患者更难达到MRD（MFC或qPCR）阴性，且MRD转阳风险更高[34]，提示对此类患者需更密切的MRD监测与更积极干预策略。同时，IKZF1改变可能影响MRD预测价值。部分研究发现伴IKZF1改变患者即使达到MRD阴性（基于qPCR或MFC检测），其复发风险仍高于无IKZF1改变的MRD阴性患者[35]，表明IKZF1改变可能通过其他机制影响疾病生物学行为，需在MRD基础上结合遗传特征进行综合风险评估。

四、伴IKZF1改变B-ALL的治疗策略进阶：从免疫治疗到移植抉择

IKZF1改变导致的生物学行为恶化（如分化阻滞、增殖失控）使其对传统化疗不敏感，这恰恰凸显了免疫治疗的核心价值。后者不依赖于传统的细胞毒性机制，而是通过调动T细胞等免疫效应细胞，实现对IKZF1改变细胞的精准清除。因此，对于这类患者，免疫治疗并非可选方案，而是克服其内在耐药性、通往移植的必经之路。

1. 免疫治疗在移植前的“桥梁”作用：对于携带IKZF1改变的B-ALL患者，在移植前实现深度缓解（尤其是MRD阴性）是改善预后的基石。新型免疫疗法在此扮演了至关重要的“桥梁”角色，其意义在于为传统化疗反应不佳或无法快速清除MRD的高危患者，提供了通往移植的新路径。

（1）CD19嵌合抗原受体T细胞（CAR-T细胞）治疗：CAR-T细胞治疗在复发/难治性（R/R）B-ALL中显示出强大的效力，研究证实其在包括IKZF1改变在内的高危遗传学亚组中，同样能诱导高比例的MRD阴性（通常通过MFC或高灵敏度qPCR评估）完全缓解，为后续移植创造宝贵机会[36]。值得注意的是，长期随访数据表明，正是这些携带IKZF1改变等高危遗传学特征的患者，在接受CAR-T细胞治疗后仍面临较高的远期复发风险，这并非否定CAR-T细胞的疗效，而是更凸显了在CAR-T细胞诱导缓解后衔接巩固性移植的必要性。这形成了一个有效的治疗闭环：免疫治疗克服初始耐药、获得缓解、移植根除残余病灶、预防远期复发。

（2）双特异性T细胞衔接器（BiTE）：BiTE如贝林妥欧单抗（blinatumomab），同样能有效清除MRD。对于不适合或不愿接受CAR-T细胞治疗的患者，贝林妥欧单抗是诱导MRD阴性（通过MFC或PCR检测），进而桥接移植的有效手段[37]。

（3）核心价值：对于IKZF1改变这一高危群体，免疫治疗的价值在于能够突破传统化疗的瓶颈，实现更深层次的分子学缓解，从而显著提高后续移植的成功率，并可能降低移植后复发风险。

2. 移植方案异基因造血干细胞移植（allo-HSCT）与自体造血干细胞移植（auto-HSCT）的权衡：移植方式的选择不应再仅基于“是否伴有IKZF1改变”这一静态指标，而应动态评估其在诱导治疗（包括化疗和免疫治疗）中的反应速度与深度（即MRD转阴时机）。

（1）allo-HSCT：allo-HSCT凭借其移植物抗白血病效应，至今仍是大多数IKZF1改变高危B-ALL患者的首选治愈性手段。大量回顾性研究证实，allo-HSCT能够部分克服IKZF1改变带来的固有高危预后，将患者的长期生存率提升至50％～60％[23],[38]。决策时需综合考量供者来源、患者年龄与合并症，以及移植相关毒性。对于经免疫治疗后仍MRD阳性，或治疗过程中MRD转阳的患者，allo-HSCT是毋庸置疑的基石。

（2）auto-HSCT：传统观念认为auto-HSCT不适用于IKZF1改变患者。然而，新的研究正挑战这一范式。关键在于严格筛选优势人群。既往研究表明，对于化疗后早期获得MRD阴性的B-ALL患者，auto-HSCT联合移植后维持治疗可获得与allo-HSCT相似的生存结局，且移植相关死亡率（TRM）更低[38]–[39]。这为在IKZF1改变患者中探索低TRM策略提供了新思路。

（3）决策核心：“MRD清除动力学”是抉择的关键。早期快速MRD转阴者，auto-HSCT是可考虑的选项；而缓解延迟或治疗过程中MRD持续阳性者，则应积极选择allo-HSCT。这种分层策略实现了疗效与毒性的最佳平衡。

五、伴IKZF1改变B-ALL患者的移植策略优化：MRD导向的个体化治疗

1. MRD导向的风险分层与移植决策：如何为伴IKZF1改变的B-ALL患者选择最合适移植策略是当前临床研究重点。基于MRD状态的风险适应治疗策略可能为此提供解决方案[40]。研究表明，达MRD阴性的时间点（即MRD清除动力学）对移植选择具重要指导价值：早期达MRD（MFC，灵敏度10⁻⁴）阴性的患者可能从auto-HSCT中获益，而较晚达MRD阴性的患者可能仍需选择allo-HSCT[39]。

该发现与回顾性队列分析的研究结果高度一致[41]：对于1个疗程化疗后经MFC检测达到MRD阴性的IKZF1改变B-ALL患者，auto-HSCT可提供与allo-HSCT相当的生存率；而对于2～3个疗程后仍需通过更高灵敏度的qPCR才能确认MRD阴性者，allo-HSCT仍显示生存优势。这种基于MRD动态变化的风险分层策略可更精准识别需强化治疗的患者，避免过度治疗及相关毒性。

2. 移植后治疗策略：从被动观察转向主动管理。鉴于IKZF1改变所赋予白血病细胞的高复发潜能和免疫逃逸能力，对于此类患者，移植后必须采取主动管理策略，而非被动观察。其目标是通过持续的免疫监视或药物干预，清除可能残存的、具有生存优势的IKZF1突变克隆，预防复发。

（1）MRD监测与抢先干预：对于移植后MRD（MFC或qPCR）阳性患者，抢先干预如供者淋巴细胞输注（DLI）或靶向药物治疗可降低复发风险。Schmid等[42]的EBMT注册研究证实，DLI在儿童ALL中可有效清除MRD、改善生存。近年来，贝林妥欧单抗等双特异性抗体也被用于移植后MRD阳性患者的抢先干预，可有效清除MRD，延缓血液学复发。

（2）维持治疗的探索：对于伴IKZF1改变患者，移植后维持治疗可能尤为重要。研究表明，采用酪氨酸激酶抑制剂、免疫调节药物或靶向抗体药物进行维持治疗，可显著降低复发风险、改善生存[43]。探索如定期输注CAR-T细胞或使用贝林妥欧单抗等作为移植后维持策略，是当前研究的前沿方向，旨在为这类高危患者提供更深层次的保护。

六、未来展望

未来研究应重点关注以下方面：①检测技术的优化与标准化：开发并普及更敏感的MRD检测技术，如NGS，以提高预后预测的准确性，并推动其在多中心的标准化应用[28],[44]。②免疫治疗应用的深化与拓展：进一步探索双特异性抗体和CAR-T细胞疗法在移植前桥接与移植后抢先干预/维持治疗中的最佳应用策略。③个体化策略的前瞻性验证：亟需开展前瞻性临床试验，验证基于“MRD清除动力学”的个体化移植决策模型在此类高危患者中的疗效与安全性[41]。④靶向药物的开发：基于IKZF1改变的分子机制，研发针对性靶向药物[45]。⑤从机制到病床：IKZF1研究的基础-临床转化路径。将IKZF1基因改变的分子机制研究成果转化为临床实践中的个体化治疗策略，是改善患者预后的关键。基础研究阐明了IKZF1改变（如显性负效应突变[14]、JAK-STAT通路协同激活[16]、表观遗传重编程[8]）导致分化阻滞、增殖失控和治疗抵抗的机制，这为“MRD清除动力学”模型提供了生物学解释。例如，显性负效应导致的深度转录调控失调，可能直接关联于患者对化疗的迟钝反应和MRD清除延迟，从而支撑了将“早期快速MRD转阴”作为筛选可能从auto-HSCT中获益患者的核心逻辑[41]。

然而，转化过程中面临诸多瓶颈：①技术准入与标准化：高灵敏度MRD检测（如NGS）和复杂分子学分型在临床常规应用尚未普及，且缺乏统一标准[28],[43]；②经济成本：CAR-T等新型免疫疗法费用高昂，限制可及性[36]；③伦理与审批：创新型个体化治疗方案的临床试验设计复杂，审批周期长。

为突破这些瓶颈，建议采取以下路径：①推动多中心验证与标准化：建立跨中心协作网络，共同验证NGS-MRD等新技术的临床效用并制定操作共识；②促进试剂国产化与医保衔接：鼓励本土研发，降低检测与治疗成本，并探索纳入医保的合理模式；③利用真实世界研究与适应性设计：积累真实世界证据，并采用适应性临床试验设计，加速有效策略的验证与获批；④加强医患沟通与教育：提升临床医师对分子标志物指导治疗的认识，同时加强对患者的宣教，提高其对个体化治疗路径的理解与依从性。

七、结论

IKZF1改变是B-ALL中一个关键的预后决定因子，它标识出一类具有内在治疗抵抗和高复发风险的疾病亚型。对于这类患者，治疗策略应是积极且前瞻性的：在诱导缓解阶段，应尽早考虑引入免疫治疗作为通往移植的桥梁；在移植决策时，不应再静态地依据IKZF1本身，而应动态结合MRD的清除速度与深度——早期快速MRD（如诱导后MFC检测阴性）转阴者，auto-HSCT是可考虑的选项，以降低治疗相关死亡；而缓解延迟或MRD持续阳性者，allo-HSCT是不可动摇的基石。移植后，则需强化MRD监测并准备积极的干预策略，包括抢先免疫治疗和维持治疗，以克服其固有的高复发倾向。未来，通过整合分子遗传学特征、MRD动态和新兴疗法，我们有望为伴IKZF1改变的B-ALL患者实现真正的个体化精准治疗，改善其远期生存。
